# Assessing the Economic Resilience of Different Management Systems to Severe Forest Disturbance

**DOI:** 10.1007/s10640-022-00719-5

**Published:** 2022-09-06

**Authors:** Thomas Knoke, Carola Paul, Elizabeth Gosling, Isabelle Jarisch, Johannes Mohr, Rupert Seidl

**Affiliations:** 1 Institute of Forest Management, Department of Life Science Systems, TUM School of Life Sciences Weihenstephan, Technical University of Munich, Hans-Carl-von-Carlowitz-Platz 2, 85354 Freising, Germany; 2 Department of Forest Economics and Sustainable Land-Use Planning, University of Goettingen, 37077 Göttingen, Germany; 3 Ecosystem Dynamics and Forest Management Group, Department of Life Science Systems, TUM School of Life Sciences Weihenstephan, Technical University of Munich, Hans-Carl-von-Carlowitz-Platz 2, 85354 Freising, Germany

**Keywords:** Disturbance economics, Engineering resilience, Present value, Willingness to pay for forestland, Forest value, Continuous cover forestry, Close to nature forestry, Tree mortality

## Abstract

Given the drastic changes in the environment, resilience is a key focus of ecosystem management. Yet, the quantification of the different dimensions of resilience remains challenging, particularly for long-lived systems such as forests. Here we present an analytical framework to study the economic resilience of different forest management systems, focusing on the rate of economic recovery after severe disturbance. Our framework quantifies the post-disturbance gain in the present value of a forest relative to a benchmark system as an indicator of economic resilience. Forest values and silvicultural interventions were determined endogenously from an optimization model and account for risks affecting tree survival. We consider the effects of differences in forest structure and tree growth post disturbance on economic resilience. We demonstrate our approach by comparing the economic resilience of continuous cover forestry against a clear fell system for typical conditions in Central Europe. Continuous cover forestry had both higher economic return and higher economic resilience than the clear fell system. The economic recovery from disturbance in the continuous cover system was between 18.2 and 51.5% faster than in the clear fell system, resulting in present value gains of between 1733 and 4535 € ha^−1^. The advantage of the continuous cover system increased with discount rate and stand age, and was driven by differences in both stand structure and economic return. We conclude that continuous cover systems can help to address the economic impacts of increasing disturbances in forest management.

## Introduction

The concept of resilience is a hot topic in ecology (Chambers et al. 2019) and has received considerable attention also in forestry (Seidl et al. 2016; Nikinmaa et al. 2020). Originally, the resilience concept comes from psychiatry, defined as the ability of people to maintain or regain mental health (Fleming and Ledogar 2008; Herrman et al. 2011; Wu et al. 2013). Since its early applications, the resilience framework has evolved considerably, and has also percolated into other fields [see for example Zampieri et al. (2020) for a recent application to crop production systems]. In ecology and economics, we broadly find two concepts of resilience (Perrings 2006): One focuses on the disturbance needed to move a system into an alternative state, while the other quantifies the speed with which a system recovers after disturbance. The first concept, introduced by Holling (1973) into the ecological literature, sees resilience as protection against a possible shift of a system into a potentially undesirable state. Examples for such regime shifts are the transition of a tropical forest into a savannah system or a savannah into a desert system. In this context, resilience describes the capacity to face disturbance and not pass a tipping point. Folke (2016) has further developed this perspective towards the adaptive capacity of systems and proposed resilience as a key property to facilitate sustainable development (Folke et al. 2002). Since then, several economic studies have built on this concept. For example, Franklin and Pindyck (2018) assessed the costs of tropical deforestation, arguing that tropical forest loss will cause adjacent tropical forest to shift to savannah ecosystems. Baumgärtner and Strunz (2014) presented an economic study on the insurance value of resilience, using a decrease in the risk premium associated with increasing resilience to quantify the contribution of ecological resilience to the economic value of ecosystems. Baumgärtner and Strunz (2014) draw on Mäler and Li (2010), who introduced the concept of resilience stock, to assess the impact of marginal changes in the resilience stock on welfare. Wu and Kim (2013), for instance, applied the idea of resilience stock to quantify resilience of pine forests prone to fire. In this context, a high resilience stock indicates a large capacity of a system to absorb disturbance, and a low risk of tipping into an undesired system state.

A second, widely applied concept of resilience uses the recovery time to a system equilibrium after disturbance as its focal metric (Pimm 1984). This recovery time concept is by far the most frequently applied quantitative approach to measure resilience in forest science (Nikinmaa et al. 2020). Some authors have introduced the term “engineering resilience” for this perspective (Peterson et al. 1998) to differentiate it from “ecological resilience” as described above. Focusing on the speed of recovery or rebound time, this concept is widely applied for instance in the analysis of the response of specific tree variables to natural disturbances (Lloret et al. 2011; Steckel et al. 2020). Most studies use the pre-disturbance system state, which frequently is conceptualized as steady or cyclic state (Peterson et al. 1998), as a benchmark to evaluate post-disturbance development. The speed-of-recovery concept has also found its way into business-economic applications. For example, Park et al. (2011) have defined economic resilience as the ability of a business to recapture lost production. However, economic studies on the speed of recovery of ecosystems after disturbance remain rare.

The perspective of economic recovery time after disturbance is highly relevant, particularly for systems where production times extend over very long periods, as is typically the case in forestry. Depending on the region considered, timber production may require rotation periods of 100 years or more. Such long-lived systems are highly vulnerable to natural disturbances, which are becoming more frequent under climate change (Seidl et al. 2017). For example, severe droughts can lead to extensive tree mortality in forests (Bréda and Badeau 2008; Senf et al. 2020) and disrupt the production process. The European drought of 2018 (Schuldt et al. 2020), for instance, has resulted in elevated levels of tree mortality also in 2019 and 2020, and affected large areas of Germany (Federal Ministry for Food and Agriculture 2021), Czechia (Hlásny et al. 2021) and beyond (Senf and Seidl 2021). Losing crop trees due to extreme disturbance before they reach their economically optimal harvest age may result in considerable economic loss. Crop trees provide regular net revenues for forest enterprises, and their loss (in combination with the need to salvage log) can lead to a substantial delay in subsequent economic returns (Nieuwenhuis 2001). Both the effect of disturbance on crop trees and the ability to recover a next generation of crop trees is strongly contingent on the silvicultural system. In this light, the economic resilience of different silvicultural systems to severe natural disturbance events is of substantial importance.

In the face of increasing natural disturbances the question of how to address such disruptions in ecosystem management has gained prominence in recent years (Grêt-Regamey et al. 2013; Seidl 2014; Seidl et al. 2017). However, compared to an increasing body of literature on ecological impacts, economic studies on resilience to natural disturbances remain scarce. Forests are notably exposed to risks from natural disturbances (Yousefpour et al. 2012). As a consequence forest economists are increasingly studying natural disturbances (Montagné-Huck and Brunette 2018) and integrating mortality-related risks into their analyses (Buongiorno and Zhou 2015; Messerer et al. 2017; Friedrich et al. 2019; Müller et al. 2019; Radke et al. 2020). However, almost all of these studies focus on disturbance resistance (Spiecker 2003; Griess and Knoke 2013; Roessiger et al. 2013), or differences between damage costs of alternative and business-as-usual forest management strategies (Hahn et al. 2021). In contrast, the rate of recovery of economic value after disturbance remains understudied in environmental economics.

Here we present a novel analytical framework to assess the economic resilience of different silvicultural systems. Our contribution has relevance beyond forestry, as it complements existing approaches, which commonly analyze a system’s capacity to absorb perturbations without changing the system’s state. Such research into the potential of ecosystems to withstand disturbances and avoid regime shifts (Mäler and Li 2010) has stimulated important economic advances (Primmer and Paavola 2021), for example concerning the insurance value of biodiversity (Baumgärtner 2007; Finger and Buchmann 2015; Bartkowski 2017; Augeraud-Véron et al. 2019; Paul et al. 2020; Schaub et al. 2020) or natural capital (Quaas et al. 2019). Focusing on recovery from disturbance may form another interesting avenue for future environmental economics research.

At the heart of our framework is an approach quantifying the economic value of a swift post-disturbance recovery. We show how recovery time influences the forest value for severe natural disturbances, i.e. in situations where no crop trees remain. A simplified optimization model that maximizes economic return is at the core of our analysis, allowing optimal management strategies to emerge based on discounted future net revenues. We here illustrate the application of our framework by comparing the economic resilience of two alternative silvicultural systems, i.e., continuous cover forestry and clear fell forestry.

Clear fell forestry concludes the production cycle (rotation) of a stand by harvesting all crop trees at the same time; the planting of young trees subsequently takes place on bare soil after all crop trees have been felled. In contrast, continuous cover forestry avoids clear felling by establishing cohorts of young trees early in stand development, and by prolonging the harvesting of crop trees, so that felling of current crop trees and the regrowth of future crop trees happens simultaneously within a stand (Kuuluvainen et al. 2012). Continuous cover forestry is frequently proposed as a strategy to address risks in forest management (Seidl et al. 2008; Hanewinkel et al. 2014). Because the next generation of trees is established before the final crop trees have reached the optimal rotation period (Soto et al. 2020; Stokes et al. 2021), this next generation of trees is able to resume timber production, should a natural disturbance remove the crop trees in the overstory. The presence of young trees could thus effectively speed up the economic recovery after disturbance, i.e. the time it takes to regain a certain level of economic forest value. However, the early initiation of a second cohort of trees requires the canopy of crop trees to be opened, so that the young tree cohort obtains enough light and water to grow. This could mean production losses and perhaps also economic losses due to harvesting of timber that has not yet reached the optimal felling age. We thus investigate two hypotheses when comparing the economic resilience of clear fell systems and continuous cover systems: (1) Establishing a young cohort of trees prior to the optimal rotation age through early timber harvesting does not significantly reduce economic returns. (2) A young cohort of trees planted prior to harvesting of the crop trees enhances the economic resilience of forests to natural disturbance.

By integrating disturbances into the economic assessment of complex management strategies and analyzing recovery rates of economic value of a forest after severe disturbance, our paper adds new dimensions to the existing body of literature. For example, previous optimization studies focused on the economic return of alternative forest management regimes to clear felling, but ignored natural disturbances (Rämö and Tahvonen 2014, 2015; Tahvonen 2015; Roessiger et al. 2016; Tahvonen and Rämö 2016; Assmuth et al. 2018, 2021; Parkatti and Tahvonen 2020). In contrast, we here integrate the influence of natural events such as wind and bark beetles using empirically derived tree survival models to simulate tree mortality and include additional scenarios of severe disturbances. In a recent study Malo et al. (2021) have shown how reinforcement learning combined with complex forest optimization can utilize Monte-Carlo-Simulations to account for natural disturbance events. Their study’s economic target variable was the willingness to pay for forestland, associated with the key assumption that only bare land remains after disturbance. Here, we go beyond this assumption, considering that saplings planted under the canopy of larger trees prior to a severe disturbance can survive such an event, because small trees are much less vulnerable to many types of disturbances than larger trees (Mason and Valinger 2013). By projecting the development of the forest’s economic value after severe disturbances, either with or without the presence of pre-established small trees, we assess the importance of pre-disturbance age structure (even-aged or uneven-aged) for the recovery of economic value. Studies on the importance of age structure for the economic resilience of a forest are to date widely absent in environmental economic research.

## An Analytical Framework to Assess the Economic Resilience of Forests

**Fig. 1:**
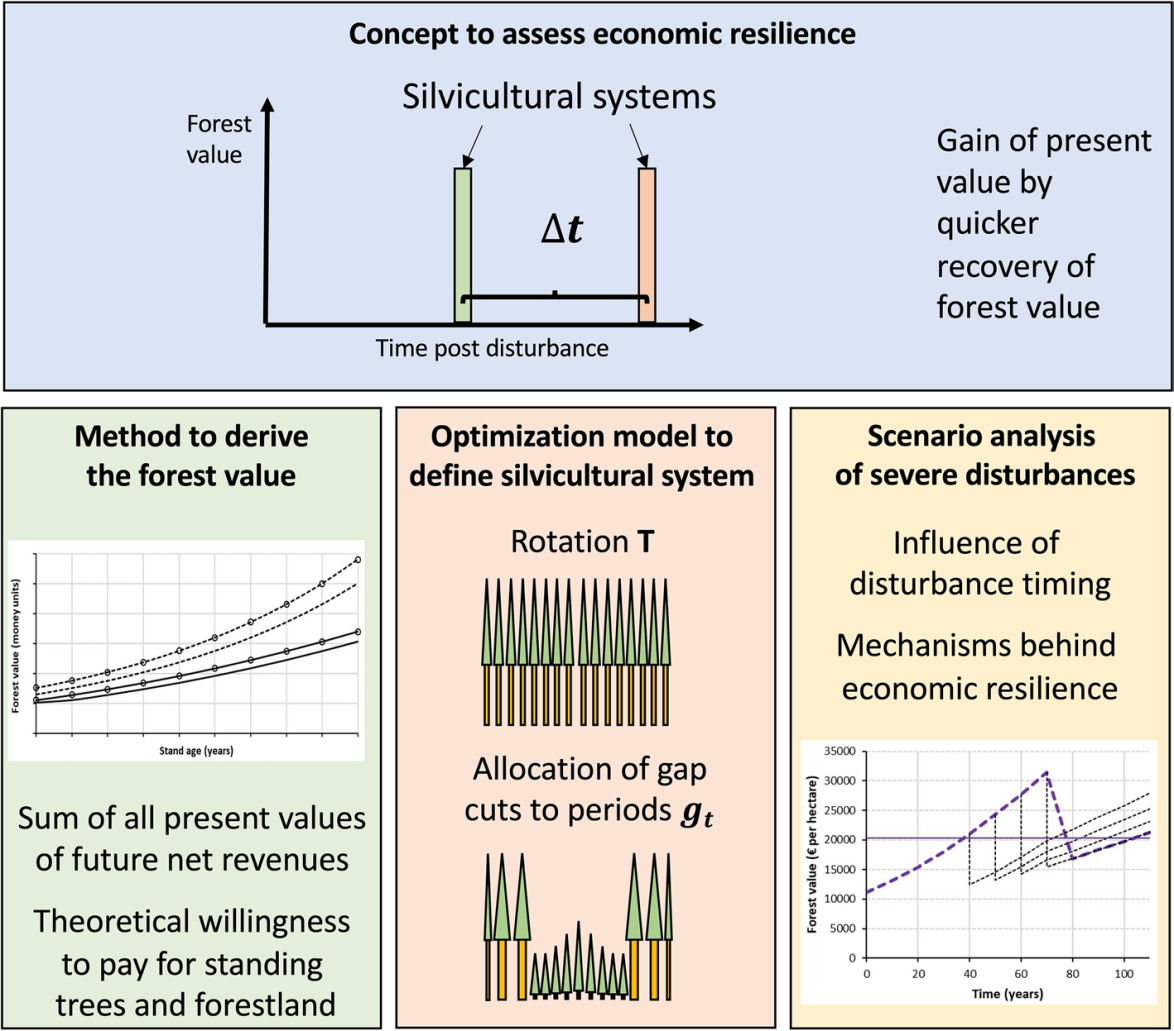
Components of our analytical framework

Our analytical framework consists of four components: A concept to assess the economic resilience of a forest, a method to derive the forest value under the influence of disturbance risk, a model to optimize the forest management, and a scenario analysis to study the mechanisms behind economic resilience (Fig. 1). Before we describe these components in detail, we introduce in the economic theory underlying our study.

We consider two different economic variables in our assessment, (1) the forest value, and (2) the economic return. The forest value, defined as all appropriately discounted net revenues expected from a given stand at age *a*, is (theoretically) the maximum price a buyer is willing to pay for such a forest stand, including the existing trees and the land itself. In contrast, the economic return considers the production period of a forest system from an age of zero onwards by always starting with bare forestland. Defined as the excess return of a stream of discounted revenues over a stream of discounted costs, we can consider the economic return as a measure of the economic performance of a certain forest system.

The economic return expected from bare forestland is referred to in the literature as the willingness to pay for forestland (WPL). WPL theory is a common framework in forest economics (Manley and Bare 2001; Kuusela and Lintunen 2019), which Faustmann first systematically documented and applied (Samuelson 2000). Moog (2020) recently described the background of this theory in the context of rotation forestry. Rotation here describes the production time from stand establishment to the harvesting of the crop trees. The WPL concept extends beyond considering only one rotation by considering an unlimited time horizon, providing that all forestry activities assumed in the first rotation cycle start from bare ground and are repeated indefinitely. The net present value concept implies that the different silvicultural strategies assessed represent marginal projects so that a preference of decision-makers for a smooth distribution of net revenues over time and/or risk aversion can be disregarded (Quaas et al. 2019; Knoke et al. 2020a). For the sake of simplicity, we here assume that only the production and marketing of timber constitute economic value.

We apply WPL theory to compare two alternative silvicultural systems. One system assumes that all crop trees are clear felled (harvested at the same time) at time *T*, with the next cohort of trees being planted after clear felling (clear fell system). The other system assumes the planting of young trees in canopy gaps created prior to the final harvesting of all crop trees. The second system simulates a transition to continuous cover forestry, here called a continuous cover system. To facilitate a realistic comparison between the two systems we used an empirical growth function for Norway spruce (Pretzsch et al. 2014) and considered age- and species-specific survival probabilities of trees (Brandl et al. 2020) in calculating the economic return as described by Staupendahl and Möhring (2011) as well as Möllmann and Möhring (2017). Building on the premise that some stands will fail to reach their scheduled rotation age, we obtained the expected values of economic returns accounting for disturbances and the required replanting of trees after post-disturbance salvage logging.

### Quantifying Economic Resilience

We were interested in the economic resilience of silvicultural systems after severe disturbance. We differentiate between such severe disturbances and background mortality, where the latter describes low severity mortality that still allows a share of the expected positive net revenues to be obtained from crop trees. Severe disturbance (e.g. caused by a strong storm or a massive bark beetle outbreak), in contrast, assumes that all expected future positive net revenues from the current generation of crop trees are lost, implying a salvage value of zero (e.g., Malo et al. 2021). Severe disturbances, which often occur across larger spatial extents, can flood the timber market with an over-supply of timber, so that forest owners cannot market their timber with positive net revenues. This situation has prevailed in many regions of Germany in 2019 and 2020, for instance, due to a severe drought starting in 2018 (Buras et al. 2020; Schuldt et al. 2020).

**Fig. 2:**
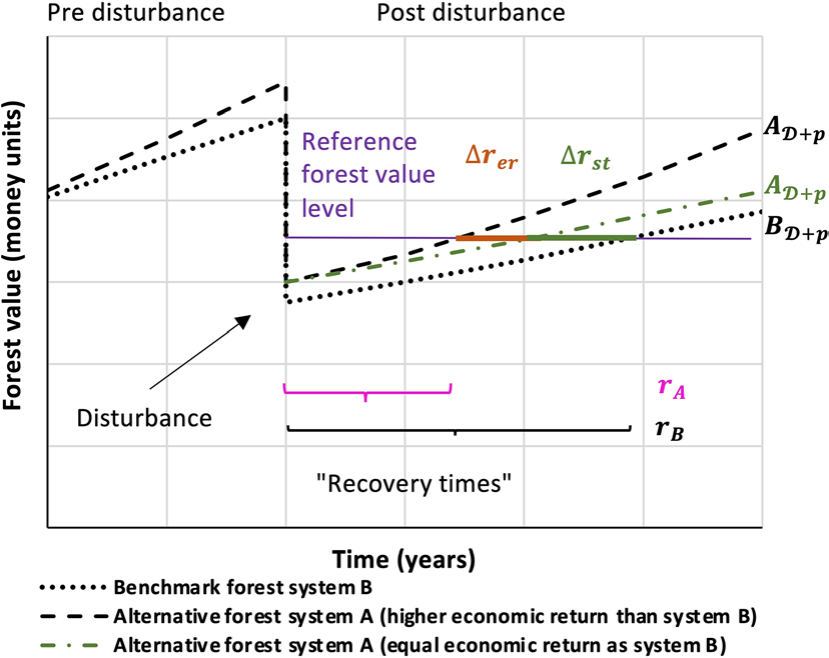
Illustration of the development of the forest value for two forest systems (with post-disturbance forest values of ${B}_{{\mathcal{D}}+p}$ and ${A}_{{\mathcal{D}}+p}$) affected by a severe disturbance, based on hypothetical curves of the development of forest value. Forest system A represents the alternative forest system, while B is the benchmark forest system. In the example shown here, system A has higher economic resilience than system B. In one of the two illustrated cases, the forest value curve for A is steeper, indicating higher economic return than system B. In the second case we assume identical economic return of A and B, but faster recovery of system A because of an effect of forest structure, i.e. a cohort of regenerating trees surviving a disturbance. The curve running parallel to the post-disturbance curve of the forest system B (symbolizing equal economic performance of both forest systems) is an example showing how the value of forest system A would develop solely based on an effect of the pre-disturbance forest structure ($\Delta {r}_{st}$). For example, a tree cohort established pre-disturbance enhances the post-disturbance forest value, when remaining unaffected by the severe disturbance. In addition to $\Delta {r}_{st}$, the post-disturbance development of the forest value of forest system A may show higher resilience by a higher economic return leading to a faster increase of the forest value ($\Delta {r}_{er}$)

Assessing the recovery from severe disturbance requires the definition of a reference state. While pre-disturbance equilibrium states are widely used in this context, this approach is not applicable here, given forest stand dynamics. In a clear fell forest, the forest value follows a cyclic trajectory over time, and even in a continuous cover forest a steady state that is persistent over longer periods of time is uncommon (O'Hara 2014). We consequently selected the clear fell strategy as reference, given its historical prominence and global application (Puettmann et al. 2015) (system B). We quantify the expected economic value of system A (continuous cover forestry) associated with recovering from severe disturbance using the benchmark forest system B as reference (schematically described in Fig. 2). For example, a faster recovery of system A would mean that a given forest value is reached earlier after disturbance under this system, associated with a higher present value of future net revenues. The time ${r}_{A}$ needed by the forest system A to achieve a given economic reference level would be smaller than that of the benchmark forest system B (${r}_{B}$, Fig. 2). In this example, system A has a higher economic resilience, which we can express by an index ($q$).

1 $$q = r_{B} \div r_{A}$$

$q$: $r_{B} \div r_{A}$; resilience index computed as the time needed to recover a certain forest value by the benchmark system divided by the time needed by the alternative system; ${r}_{B}$: time the benchmark system needs to achieve a specific level of the forest value; ${r}_{A}$: time the alternative system needs to achieve a specific level of the forest value.

The difference in recovery time derives from the index $q$ as:

2 $$\Delta r={r}_{B}-{r}_{A}={r}_{B}\left(1-\frac{1}{q}\right)$$

We assess the economic effect of the difference in recovery time as the difference in present value of future net revenues, which we can express as a fraction of the forest value ($\varOmega$):

3 $$\varOmega =1-{\beta }^{\Delta r}$$

$\varOmega$: relative change of economic value when achieving the forest value $\Delta r$ years earlier/later than a benchmark forest; $\Delta r$: difference in recovery time, $\Delta r={r}_{B}-{r}_{A}$; $\beta$: discount factor, $\beta =\frac{1}{1+i}$, with $i=0.015$ assumed as default discount rate.

**Fig. 3:**
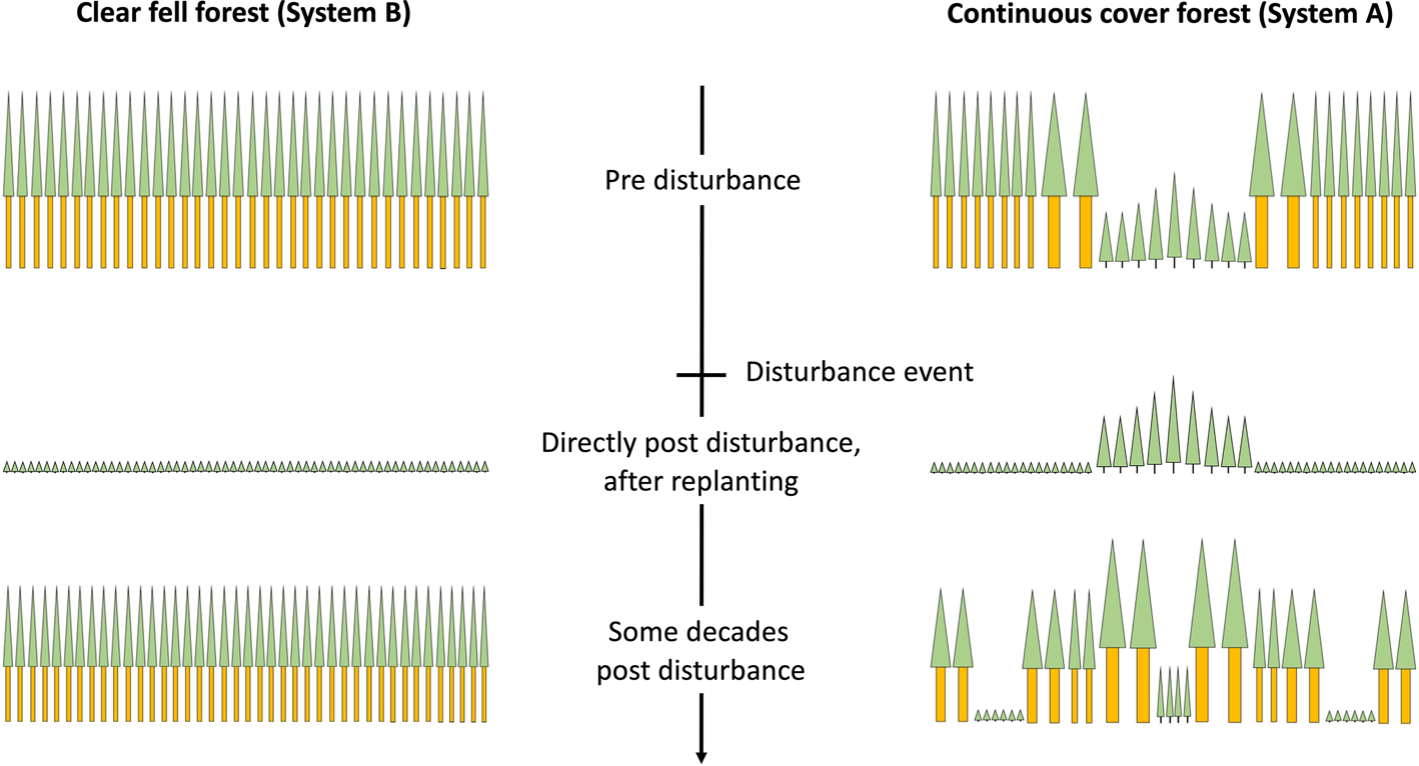
Illustration of the concepts of economic return- and structure-based differences in economic resilience. Economic return-based effects refer to the future economic return of the forest developing post-disturbance; for example, economic return could be boosted by accelerated growth of single trees induced by lower stand density, leading to larger, but less numerous trees (as displayed for the continuous cover forest). Structure-based effects refers to the differences in present value post disturbance resulting from differences in pre-disturbance forest structure (e.g., the presence of a second tree cohort that is not affected by a disturbance)

Differences in the recovery time can result from two different processes (Fig. 3). One is related to the difference in the forest value immediately after the disturbance event ($\Delta {r}_{st}$), which mainly depends on the pre-disturbance forest structure and size-dependent susceptibility to disturbance. If previously established young trees remain unaffected by a disturbance (as is the case e.g., for wind and bark beetle disturbances, which exclusively affect pole-sized trees and larger), the forest value post disturbance is higher compared to when all trees of a single layer of crop trees are affected by disturbance. The second effect results from possible differences in the expected economic return (described below) between both forest systems ($\Delta {r}_{er}$), where superior economic performance leads to a faster increase in the forest value post disturbance.

4 $$\Delta r=\Delta {r}_{st}+\Delta {r}_{er}$$

$\Delta {r}_{st}$: faster recovery because the value of silvicultural system A immediately after the disturbance is higher than that of system B. This is an effect of the pre-disturbance forest structure, because forest type A already has established a regenerating tree cohort, whereas forest type B has to resume production from age 0; $\Delta {r}_{er}$: faster recovery because of higher economic performance. Higher economic returns may result from the growth response of trees in the post-disturbance forest when stand density is reduced after establishing canopy gaps and planting young trees; the remaining canopy trees can grow faster than trees growing in stands of higher density (see Fig. 3).

For the change in present value, we write:

5 $${\Delta v}_{{\mathcal{D}}+p}={A}_{{\mathcal{D}}+p}\varOmega$$

${\Delta v}_{{\mathcal{D}}+p}$: change of present value associated with the resilience of system A due to a difference in recovery time at current post-disturbance time *p* [all values here in € per hectare]; ${A}_{{\mathcal{D}}+p}$: post-disturbance forest value of forest system A at stand age $a$, when ${\mathcal{D}}$ represents the time of disturbance; ${A}_{{\mathcal{D}}+p}$ comprises all discounted future net revenues over an unlimited time, starting from post-disturbance time *p* onwards.

We find the corresponding recovery times of the forest systems (${r}_{A}, {r}_{B}$) to achieve a defined forest value, using Eqs. (6) and (7).

6 $$\left\{\begin{array}{l}{A}_{{\mathcal{D}}+{r}_{A}}={A}_{0}+{a}^{{\prime}}{r}_{A} \\ {B}_{{\mathcal{D}}+{r}_{B}}={B}_{0}+{b}^{{\prime}}{r}_{B}\end{array}\right.$$

7 $$\left\{\begin{array}{l}{r}_{A}=\frac{{A}_{{\mathcal{D}}+{r}_{A}}-{A}_{0}}{{a}^{{\prime}}} \\ {r}_{B}=\frac{{B}_{{\mathcal{D}}+{r}_{B}}-{B}_{0}}{{b}^{{\prime}}}\end{array}\right.$$

${A}_{{\mathcal{D}}+{r}_{A}},{B}_{{\mathcal{D}}+{r}_{B}}$: post-disturbance forest value (after severe disturbance) of the forest systems; forest values represent all discounted net revenues over an unlimited time, starting from a given post-disturbance time, either ${\mathcal{D}}+{r}_{A}$ or ${\mathcal{D}}+{r}_{B}$, of the forest stands [all values here in € per hectare]; ${A}_{0}, {B}_{0}$: value of the silvicultural systems at the beginning of the post-disturbance period considered; ${a}^{{\prime}}, {b}^{{\prime}}$: annual change in the forest value of silvicultural system A (${a}^{{\prime}}$) and B (${b}^{{\prime}}$).

The change of present value ${\Delta v}_{{\mathcal{D}}+p}$ quantifies the economic advantage/disadvantage resulting from changes in recovery post disturbance.

In our calculations for both economic variables, forest value and economic return, we distinguish between severe disturbances and background mortality. Background mortality represents the frequency and intensity of historical tree mortality, which we consider in our expected values for both economic variables. Background mortality refers to events for which a probability is predictable, so that we can anticipate the proportion of a forest’s area likely to be affected by such background mortality using statistical survival models. We use these probabilities to obtain weighted forest values and economic returns, which we consider as expected values. Statistical models, for example published by Brandl et al. (2020) can deliver information to quantify such background mortality rates. They do, however, fall short in capturing extreme events with severe economic consequences [see Knoke et al. (2021) for more details]. We model the net revenues after disturbance based on a factor $\gamma$, which is the proportion of the regular stumpage value (i.e., the net revenue after harvesting and marketing the timber) that can be achieved after disturbance. While we assume zero net revenues from salvage logging after severe disturbance in our analysis ($\gamma =0$) (Knoke et al. 2021), background mortality events commonly result in reduced but still positive net revenues ($\gamma =0.5$), despite lower timber prices and higher logging costs (Möllmann and Möhring 2017). In the following, we first describe the calculation of the expected forest value and economic return and second the simulation of discrete severe disturbance events.

### Calculating the Expected Forest Value

#### Forest Value of the Benchmark Forest System

To obtain the forest value for our benchmark forest system B under the business-as-usual risk (called background mortality in our study) we write:

8 $${B}_{a}={C}_{a}+{D}_{a}+{F}_{a,D}$$

${B}_{a}$: expected economic value of the benchmark forest system, considering background mortality, but no severe disturbance events [all values here in € per hectare]; $a$: age of the forest stand; ${C}_{a}$: present value of all net revenues from crop trees and trees planted after the harvest of crop trees on the share of forest area surviving until *T* (estimated by the stand’s survival probability), considered for a stand of age *a*, and discounted over *T–a*. Crop trees are trees that would reach the scheduled production age without disturbance; ${D}_{a}$: present value of all future net revenues still occurring in association with background mortality, expected for a stand of age *a*, and discounted over *t–a*; ${F}_{a,D}$: sum of forest values of tree cohorts previously planted on areas affected by background mortality and salvaged in each period until *a*.

With:

9 $${C}_{a}={S}_{T}\left({h}_{T}+{L}_{T}\right){\beta }^{T-a}$$

10 $${D}_{a}=\sum_{t=a+l}^{T-l}{S}_{t}{\pi }_{t}\left(\gamma {h}_{t}+{L}_{T}\right){\beta }^{t-a}$$

11 $${F}_{a,D}=\sum _{t=0}^{a}{S}_{t}{\pi }_{t}{F}_{a-t}$$

12 $${S}_{T}+\sum_{t=a+l}^{T-l}{S}_{t}{\pi }_{t}+\sum_{t=0}^{a}{S}_{t}{\pi }_{t}=1$$

$T$: age at the end of the rotation; $t$: time in years; *t* assumes 10-year steps; e.g. $t=0, 10, 20, 30, 40,\dots , T-l$; $l$: length of periods considered; 10 years in this study; ${{S}_{T};S}_{t}$: survival probability incorporated as the proportion of a stand’s area covered by crop trees of a maximum density surviving until *T* and *t*, respectively; ${h}_{T}; {h}_{t}$: net revenues from harvesting and selling the timber of all crop trees (stumpage value) at rotation age *T* and *t*, respectively [€ per hectare]; stumpage values result from multiplying the standing timber volume in cubic meters at $T$ or $t$ with the corresponding net timber prices (see Table 1 for the information sources used); ${h}_{T}=volum{e}_{T}\cdot pric{e}_{T}$; ${h}_{t}=volum{e}_{t}\cdot pric{e}_{t}$; ${L}_{T}$: anticipated economic return of future tree generations, newly planted with establishment costs ${C}_{0}$ after background mortality events on the salvaged areas or on regularly harvested areas, given a forest stand under the benchmark management system depending on *T*; i.e. the sum of all discounted net revenues anticipated over an unlimited time, also known as willingness to pay for bare forestland [€ per hectare]; $\sum_{t=a+l}^{T-l}\left(\cdot \right)$: captures all future periods from age *a* + *l* onwards, when background mortality will still occur; ${{S}_{t}\pi }_{t}$: area proportion salvaged after stand failure due to background mortality on which young trees will be planted; ${\pi }_{t}$: hazard rate at *t*, ${\pi }_{t}=\frac{{S}_{t}-{S}_{t+l}}{{S}_{t}}$; $\gamma$: damage losses; proportion of the regular stumpage value that can be achieved for damaged timber in a disturbance event; $\sum_{t=0}^{a}\left(\cdot \right)$: captures all periods up until age *a*, where stand failure due to background mortality has already occurred and a new forest has developed value over *a–t*; ${F}_{a-t}$: forest value [€ per hectare] represented by trees planted on areas salvaged after background mortality events at time *t*, subject to $F_{a} : = B_{a} \forall a$. The constraint means that young trees planted after background mortality events that occur immediately after planting the initial stand (which are the trees planted at the oldest replanted areas) will have the same age and area-related forest value as the whole stand.

The anticipated economic return of future tree generations (${L}_{T}$) integrates all discounted net revenues occurring after the harvest (regular or salvage) of crop trees considered over an unlimited time.

#### Forest Value of the Alternative Forest System

For the alternative forest system A, we assume a time horizon *H* over which forest managers harvest all current crop trees to convert the forest into an uneven-aged, continuous cover structure. In addition, we use a set of cuts $\omega =\left\{{g}_{t=z};\dots ;{g}_{H-l}\right\}$, describing the relative size of partial harvests of crop trees in different periods. To capture the discounted net revenues associated with partial harvests (gap cuts) we introduce two additional components to the calculation. These account for net revenues from (1) felling crop trees to create gaps in the future, including the subsequent revenues from young trees planted in the gaps (${G}_{a,\omega }$) and (2) from trees already planted on gap areas before achieving age $a$ (${F}_{a,\omega }$). The aggregate forest value is based on five components (see details in “Appendix 2”):

13 $${A}_{a}={C}_{a}+{G}_{a,\omega }+{D}_{a}+{F}_{a,\omega }+{F}_{a,D}$$

${A}_{a}$: expected economic value of the alternative forest system A, considering background mortality, but no severe disturbance events [all values here in € per hectare]; ${C}_{a}$: present value of all net revenues from crop trees remaining until *H* and trees planted after the harvest of the last crop trees, considered for a stand of age *a*, thus discounted over *H–a*; ${G}_{a,\omega }$: sum of present values associated with future partial harvests, consisting of all discounted net revenues from crop trees harvested to create gaps and from young trees planted after the harvest of crop trees over an unlimited time, for a stand of age *a*, thus discounted over *t–a*; ${D}_{a}$: present value of all net revenues occurring from background mortality at any future *t*, considered for a stand of age *a*, and therefore discounted over *t–a*; ${F}_{a,\omega }$: sum of forest values of tree cohorts planted after partial harvests in gaps at any *t* until reaching *a*; ${F}_{a,D}$: sum of forest values of tree cohorts previously planted on areas affected by background mortality events and salvaged in each period until *a*.

### Economic Optimization of Silvicultural Strategies

To assess the resilience of an alternative forest system against a benchmark system, we require information on the management of both forest systems. Such information includes the optimal rotation time (benchmark system B) and when and at which rate to establish young trees after the partial harvest of crop trees (alternative system A). In our framework, this information is derived endogenously via an optimization approach, which maximizes the economic return.

#### The Benchmark Forest System

We formulate a classical objective function for the benchmark forest system B (Eq. 14), assuming that the harvest time for all crop trees (*T*) is the same. *T* is the rotation period and the only decision variable.

14 $${R}_{T}=-{C}_{0}+{C}_{T}+{D}_{T}$$

${R}_{T}$: economic return of the benchmark forest system, when starting from bare ground, considering background mortality events [all values in € per hectare]; ${C}_{0}$: establishment costs for tree planting; ${C}_{T}$: present value of all net revenues from crop trees and from all trees planted after the harvest of crop trees over an unlimited time on the proportion of a forest’s area surviving until *T*; ${D}_{T}$: present value of all net revenues accumulated over *T* associated with salvage logging due to background mortality events, including the economic return from all young trees planted on the salvaged areas over an unlimited time.

Based on Eq. (14), we can search for the rotation *T* that maximizes the economic return. For details, see “Appendix 1”.

#### The Alternative Forest System

To obtain the economic return of forest system A we relax the requirement of the harvest age being the same for all crop trees. We consider the opportunity of partial harvests to create gaps at different periods for the establishment of young trees (new tree cohorts) associated with a simultaneous reduction in the density of remaining crop trees. Due to the reduced density the remaining crop trees will grow faster (Pretzsch 2020) (Figs. 3 and 8a), which we consider explicitly in our optimization. The economic return for the alternative forest system consists of four components (Eq. 15).

15 $${R}_{\omega }=-{C}_{0}+{C}_{H}+{G}_{\omega }+ {D}_{H}$$

${R}_{\omega }$: expected economic return of the alternative forest system A, considering background mortality events [all values here in € per hectare]; ${C}_{0}$: establishment costs for tree planting; ${C}_{H}$: present value of all net revenues from crop trees and trees planted after the harvest of crop trees over an unlimited time on the proportion of a forest’s area surviving until *H*; ${G}_{\omega }$: present value of partial harvests consisting of all discounted net revenues from crop trees harvested in gaps and from young trees planted after the harvest of crop trees over an unlimited time; ${D}_{H}$: present value of all net revenues accumulated over the horizon *H* associated with salvage logging due to background mortality events, including the economic return from all young trees planted on the salvaged areas over an unlimited time.

Utilizing a non-linear optimization algorithm, we maximize Eq. (15) by an appropriate allocation of gap cuts of different sizes over *H* to establish regeneration of young trees. For details, see “Appendix 1”.

### Quantifying the Economic Consequences of Severe Disturbances

To evaluate the economic resilience of different silvicultural strategies we simulate several discrete severe disturbance events. We assume the disturbance only to affect the current generation of crop trees (i.e., the big trees, which is in line with patterns of wind and bark beetle disturbance observed across Europe). We further assume that achievable timber prices are reduced after disturbance, and that this together with enhanced logging costs results in zero net revenues from salvaging ($\gamma =0$). We set the age of 30 as the earliest possible age to initialize the establishment of young trees via gap cuts in the alternative silvicultural system A, and simulated severe disturbance events for stands ages from 40 to 70 years.

For the post-disturbance development of forest value after a severe disturbance, we write:

16 $${B}_{{\mathcal{D}}+p}={S}_{\mathcal{D}}{B}_{p}+\sum_{t=0}^{{\mathcal{D}}-l}{S}_{t}{\pi }_{t}{B}_{p+{\theta }_{t}}$$

17 $${S}_{\mathcal{D}}+\sum_{t=0}^{{\mathcal{D}}-l}{S}_{t}{\pi }_{t}=1$$

18 $${A}_{{\mathcal{D}}+p}={S}_{\mathcal{D}}{A}_{p}+\sum _{t=0}^{{\mathcal{D}}-l}{\left(1-{g}_{t}\right)}S_{t}^{u}{\pi }_{t}{A}_{p+{\theta }_{t} }+\sum _{t=z}^{{\mathcal{D}}-l}{g}_{t}{S}_{t}^{u}{A}_{p+{\theta }_{t} }^{{\prime}}$$

19 $${S}_{\mathcal{D}}+\sum _{t=0}^{{\mathcal{D}}-l}{\left(1-{g}_{t}\right)}S_{t}^{u}{\pi }_{t}+\sum _{t=z}^{{\mathcal{D}}-l}{g}_{t}{S}_{t}^{u}=1$$

With:

20 $$System\,\, B\left\{\begin{array}{l}{\theta }_{t}=D-t\,\,\, if\,\, D-t+p<T \\ {\theta }_{t}=D-t-T\,\,\, if\,\, D-t+p\ge T\end{array}\right.$$

21 $${System\,\, A: \theta }_{t}={\mathcal{D}}-t$$

${B}_{{\mathcal{D}}+p}$: current post-disturbance economic value of the benchmark forest system B [€ per hectare]; ${A}_{{\mathcal{D}}+p}$: current post-disturbance economic value of the alternative forest system A [€ per hectare]; ${\mathcal{D}}$: time of severe disturbance; $p$: current time post disturbance; ${{S}_{\mathcal{D}};S}_{t}$: survival probability incorporated as the proportion of a forest’s area covered by crop trees of a maximum density surviving until ${\mathcal{D}}$ and $t$, respectively; ${B}_{p}$: forest value of the benchmark forest system at current post-disturbance time *p* when planted after severe disturbance [€ per hectare]; ${A}_{p}$: forest value of the alternative forest system at current post-disturbance time *p*, when planted after severe disturbance [€ per hectare]; $t$: pre-disturbance time in years, when reestablishment of trees has occurred on areas salvaged due to background mortality events; *t* assumes 10-year steps; e.g. $t=0, 10, 20, 30, 40,\dots ; l$: period length; 10 years in this study; ${\pi }_{t}$: hazard rate at $t$; ${S}_{t}^{u}$: survival probability until $t$ corrected by the area of gap harvest activities; ${{S}_{t}\pi }_{t}$: proportion of area salvaged after background mortality events, on which young trees have been planted at $t; {B}_{p+{\theta }_{t}}$: forest value of the benchmark forest system, planted at time $t$ due to salvage logging after background mortality events (prior to the severe disturbance); ${\theta }_{t}$: difference between the age of a cohort planted at time $t$ due to salvage logging after background mortality events (prior to the severe disturbance) and the age of the post-disturbance stand *a*; ${g}_{t}$: proportion (as a fraction of the area occupied by crop trees) of a gap cut carried out at time $t; {A}_{p+{\theta }_{t}}$: forest value of the alternative forest system, planted at time $t$ due to salvage logging after background mortality events (prior to the severe disturbance); $z$: time when simulating the first gap felling and subsequent establishment of young trees, for which we assume a minimum age of 30; ${A}_{p+{\theta }_{t}}^{{\prime}}$: forest value of the alternative system, planted after a partial harvest operation (gap cut) at time $t$ (prior to severe disturbance); $T$: stand age when achieving the rotation time.

To analyze the contribution of different mechanisms to economic resilience (cf. Fig. 3) in more detail we study three scenarios over a timeframe of one forest owner’s generation (assumed at 40 years):

(I) Economic return-based resilience: We simulate a severe disturbance of a stand with identical pre-disturbance structure of systems A and B.
(II) Structure-based resilience: Here we assume forest stands with different pre-disturbance forest structures. We force the alternative forest management system to already establish young trees at an earlier age compared to the optimal trajectory, to enhance the vertical structure of the stand, accepting a possible reduction in economic return. While being economically “sub-optimal”, forest system A already contains two cohorts of young trees when the severe disturbance hits. The scenario thus particularly quantifies the economic effect of high structural diversity.
(III) Combined economic return- and structure-based resilience: Management of the alternative forest system is optimal regarding economic returns.

### Material

To illustrate our approach we simulated a Norway spruce forest in Central Europe. We summarize the data used in our simulations in Table 1.

**Table 1:** Information used for simulating the economic resilience of a Norway spruce forest in Central Europe; partly adopted from Knoke et al. (2020b)

Information	Description
Background mortality based on survival probabilities	Data on tree survival adopted from Brandl et al. (2020). Assumed climate data: temperature maximum for the warmest month 19 °C, mean annual temperature 7.4 °C, minimum temperature of the coldest month − 5.6 °C, precipitation sum of the warmest quarter 270 mm. This corresponds to average values of the data used by Brandl et al. (2020)
Growth and yield	A growth function was adopted from Pretzsch et al. (2014), returning the $volum{e}_{t}$ of the standing timber of an undisturbed stand at a given age $t$
Stumpage prices	Net timber $price{s}_{t}$ adopted from Paul et al. (2019)
Default reduction of the stumpage value of damaged timber after a disturbance event ($\gamma$)	Assumptions based on Dieter (2001) as well as Möllmann and Möhring (2017) for background mortality events, and on Knoke et al. (2021) for severe disturbance events
Reduction of economic return for tree cohorts planted in gaps	We combined a statistical model of relative light availability in response to stand density, published by Kateb (2006), with a statistical model on the growth of young trees under reduced light availability available from Petriţan et al. (2009). Figure 8a in the “Appendix” describes the resulting relationship in more detail
Growth response of remaining trees after partial harvest	We used data from a thinning experiment analyzed by Härtl et al. (2010) to derive a factor quantifying the relative increase of the trees’ value growth under different stand densities. Figure 8b shows the resulting relationship

A growth function for Norway spruce based on extensive empirical data, published by Pretzsch et al. (2014), calculates the standing timer volume in cubic meters ($volum{e}_{t}$) for a given stand age $t$, with the change from year to year corresponding to timber growth rate. Multiplying the standing timber volume with net timber prices ($pric{e}_{t}$), extracted from Paul et al. (2019), results in the stumpage value of standing timber (${h}_{t}$) in an undisturbed forest. Survival probabilities (${S}_{t}$) and hazard rates (${\pi }_{t}$) were adopted from Brandl et al. (2020) to account for background mortality.

## Results

In this chapter, we illustrate the application of our analytical framework by comparing the economic disturbance resilience of a continuous cover forest against the benchmark of a clear fell forest. We first describe the results of our economic optimization for both silvicultural systems, then report their resilience, and finally investigate the different mechanisms behind the economic resilience of the two systems.

### Economically Optimal Management

While the optimal rotation was 70 years for the clear fell system (system B), allowing for the creation of gaps to establish young trees before the age of 70 led to a slightly higher economic return in system A (Table 2). The economic return of the continuous cover system (system A) was approximately 10% greater than that of the clear fell system. Instead of clear felling all crop trees, the optimal continuous cover system suggests six gap cuts at different periods, starting with age 60. Consequently, some trees grow as old as 110 years in the continuous cover system, instead of the maximum of 70 years in the clear fell system. We note that the optimization for the continuous cover system (Eq. 15) also converges to a clear felling regime (with identical rotation as obtained for the clear fell system) when the faster growth of remaining trees after gap cuts is neglected. Accounting for the biological growth response to partial cutting is thus essential for deriving realistic optimal management strategies.

**Table 2:** Results of the optimization of economic returns

Age	Silvicultural system A (continuous cover), economic return of 10,227 € per hectare							Silvicultural system type B (clear fell), economic return of 9225 € per hectare						
	Cohorts [ha]	Timber [only crop trees, m^3^]		Net revenues [€]		Economic return young trees [€]		Clearing at *T* [ha]	Timber [only crop trees, m^3^]		Net revenues [€]		Economic return young trees [€]	
		R	*S*	R	*S*	R	*S*		R	S	R	S	R	S
0				− 2000			*541*				− 2000			*488*
10							*541*							*488*
20			*2*		*14*		*679*			*2*		*14*		*612*
30			*9*		*107*		*691*			*9*		*107*		*623*
40			*14*		*272*		*681*			*14*		*272*		*614*
50			*20*		*497*		*657*			*20*		*497*		*593*
60	0.466	306	*5*	10,741	*183*	4707	*162*			*21*		*705*		*564*
70	0.067	58	*1*	2334	*37*	681	*84*	0.568	384		17,041		5242	
80	0.022	26	*0.4*	1135	*20*	223	*54*							
90	0.028	45	*0.2*	2189	*7*	290	*19*							
100	0.007	16		799	*2*	71	*9*							
110	0.007	25		1266		76								

*R*: timber harvests or net revenues from regular harvest operations; *S*: timber harvests or net revenues from salvage logging (γ = 0.5). All values are per hectare [ha] and net revenues are undiscounted values. For the economic return of young trees we report present values over unlimited time; discounting (*i* = 0.015) and subsequently summing the resulting present values. The area of silvicultural system B harvested at rotation *T* (${S}_{T}=0.568$, which can be interpreted as share of the total area, or as hectares for an idealized stand area of 1 hectare) corresponds to the survival probability up to this age. It shows that we simulated previous stand failure due to background mortality events, subsequent salvage logging and reestablishment for 43.2% of the area ($\sum_{t=0}^{T-p}{S}_{t}{\pi }_{t}=0.432$)

**Fig. 4:**
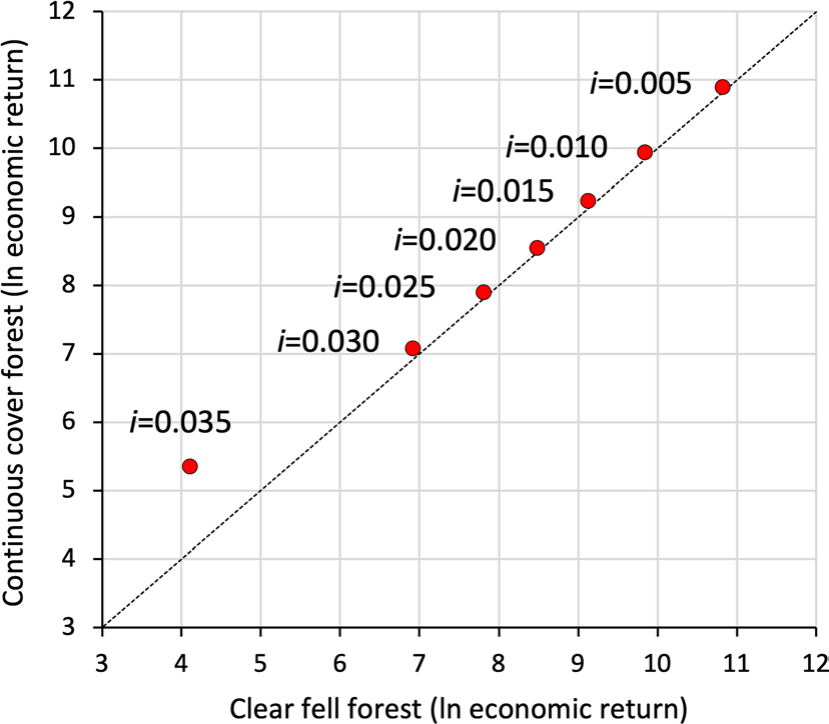
Relationship between (ln-transformed) economic return of the continuous cover and the clear fell system for various discount rates (*i* is the discount rate). The dashed line shows the 1:1 line, indicating similar performance between the two systems

Our optimization model reproduced general patterns also found in previous studies. For example, greater economic return of uneven-aged forest management compared to clear felling is found in other economic studies (see Sect. 4). Furthermore, our model realistically indicates the tendency of increasing advantages of continuous cover management with increasing discount rate (Fig. 4). For example, the continuous cover system shows 16% (discount rate 3%) or 243% (discount rate 3.5%) higher economic return than the clear fell system, while the advantage is on average about + 8% for discount rates lower than or equal to 2.5%. Our optimized management systems thus provide a plausible study case for analyzing the economic resilience of the two silvicultural systems to natural disturbance.

### Economic Resilience to Forest Disturbance

**Fig. 5:**
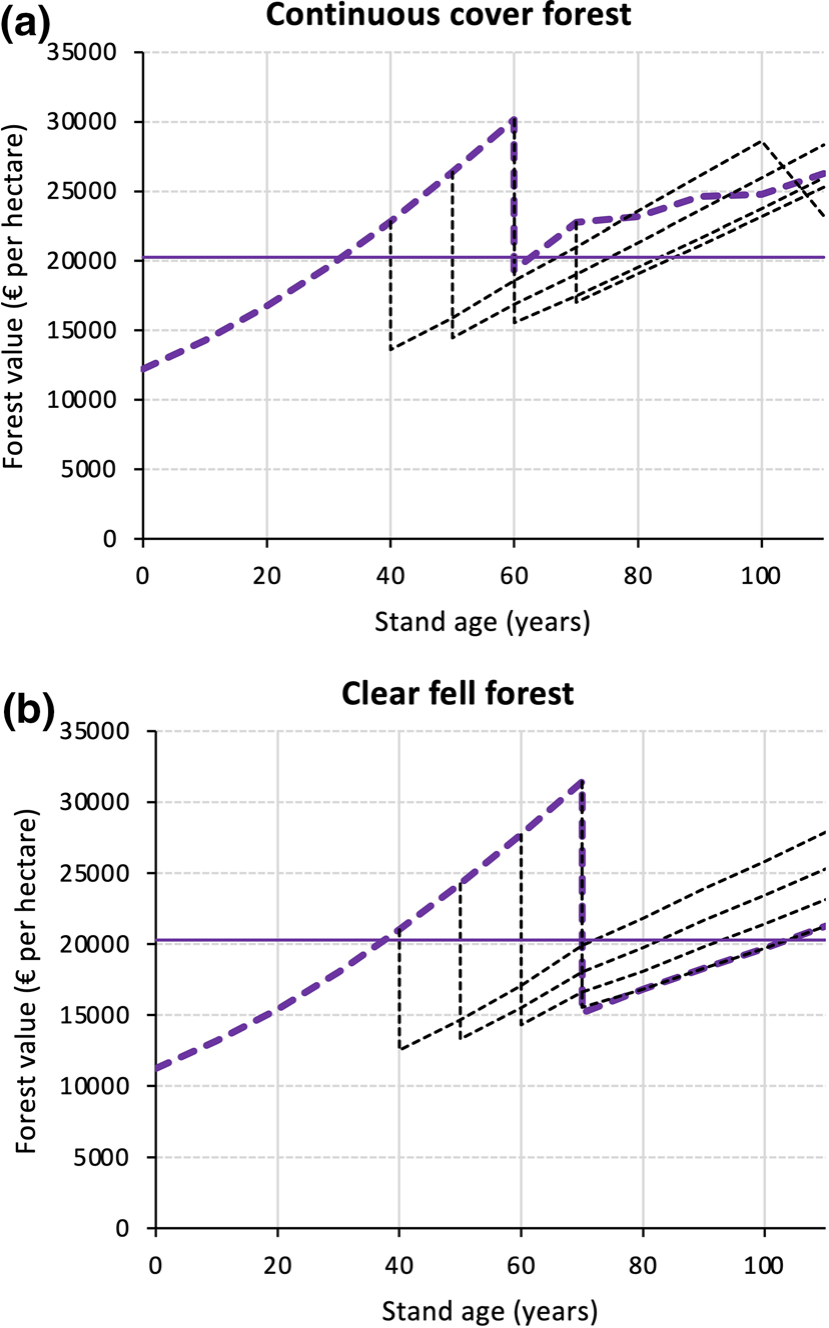
Recovery of forest value after a severe disturbance event affecting the stand at different stand ages (black dashed lines). Dashed purple lines describe the undisturbed development. **a** Alternative silvicultural system (continuous cover). **b** Benchmark silvicultural system (clear fell). The reference forest value (solid horizontal purple line) represents the average forest value of the clear fell system over a rotation period

The continuous cover system generally had higher economic resilience to disturbances than the clear fell system. However, the economic advantage of the continuous cover system was contingent on the age at which severe disturbance strikes a stand. For example, when severe disturbance affects a stand at age 70, the continuous cover system recovers 17 years faster to an economic reference level (here: the average forest value of the clear fell system over the rotation period) than the clear fell system, resulting in an economic advantage of 22.4% (Fig. 5, Table 3). While a disturbance in older stands leads to the loss of crop trees, the cohort of young trees planted in gaps remains for the continuous cover system, and already represents a notable forest value. Consequently, the continuous cover system is faster in regaining forest value compared to the clear fell system, indicating that the pre-disturbance forest structure represents an important component of economic resilience.

If we consider severe disturbances in relatively young forests (i.e., at stand ages between 40 and 60 years), the post-disturbance performance in terms of economic return exerts strong control over the recovery of economic value after disturbance. Before age 60, system A has not yet undergone partial harvesting, so the structure of systems A and B are identical. This negates any structural advantage that system A might have over system B. In this case, any difference between the (post-disturbance) economic return of the two systems results from the fact that remaining trees will grow faster after future partial cutting in the stands established post disturbance in system A (see Fig. 3). Consequently, for severe disturbances in younger stands, the economic advantage of the continuous cover system roughly equals its economic return advantage driven by changes of tree growth (Table 3).

**Table 3:** The economic value of disturbance resilience

Disturbance at age ${\mathcal{D}}$ [years]	Time to achieve a forest value of €20,275 per hectare (average value of the clear fell system) [years]		Resilience quotient $q$	Relative economic value of accelerated recovery $\varOmega \cdot 100$	Present value of accelerated recovery ${\Delta v}_{{\mathcal{D}}+p}$
	System A	System B		[%]	[€ per hectare]
40	27	33	1.22	8.5	1733
50	25	33	1.32	11.3	2277
60	23	34	1.48	15.1	3063
70	16	33	2.06	22.4	4534

### Mechanisms of Economic Resilience

**Fig. 6:**
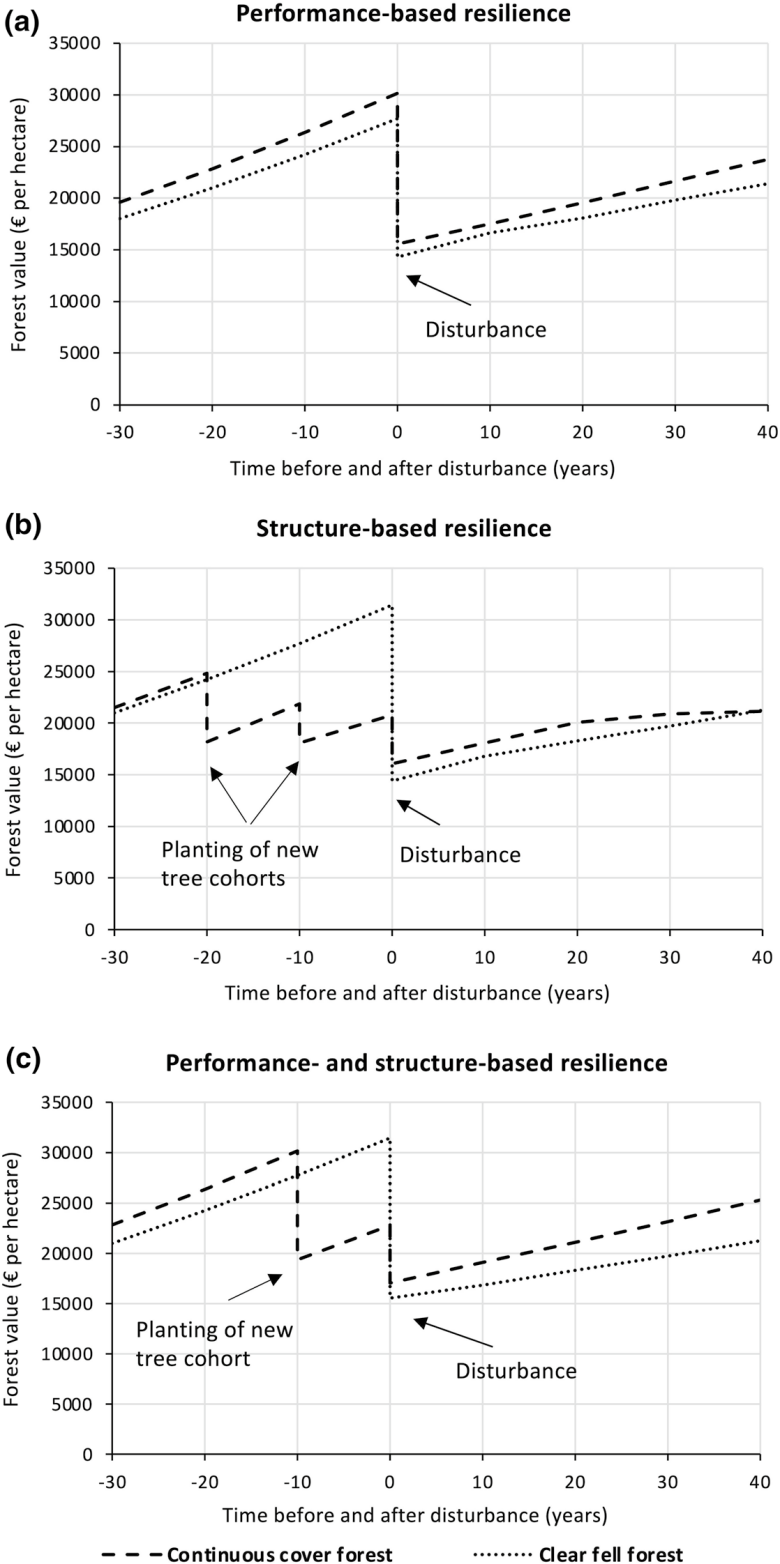
Three scenarios illustrating the different mechanisms driving the economic resilience to disturbance. **a** Disturbance at age 60. **b** Disturbance at age 70, when partial harvesting started already at age 50 in the continuous cover system. **c** Disturbance at age 70, management of both silvicultural systems is optimal with regard to their economic return

Scenario (I) indicates that the continuous cover system recovers specific levels of forest value faster than the clear fell system after disturbance at age 60, because of its higher economic return (economic return-based resilience) (Fig. 6a). Scenario (II) reveals that establishing young trees already at the age of 50 decreases the economic return of the continuous cover system by 7%. Yet, the overall economic return of the continuous cover system is still marginally better than that of the clear fell system. In this scenario, the establishment of two cohorts of young trees prior to disturbance allows for a faster post-disturbance recovery of the forest value under the continuous cover system (structural resilience). This effect persists for several decades but diminishes 40 years post disturbance, where the forest value of the continuous cover and the clear fell systems converged (Fig. 6b). Scenario (III) illustrates the combined effect of economic return- and structure-based resilience, resulting in the highest overall advantage in economic resilience of the continuous cover forest compared to the clear fell system (Fig. 6c).

The structure-based resilience mechanism enhances the recovery of forest value specifically in the two decades after disturbance, and can even lead to slightly higher economic advantages than economic return-based resilience (Table 4). The economic gain in present value associated with a higher resilience of a continuous cover system compared to a clear fell system can amount to up to 25% over 40 years post disturbance, when economic return- and structure-based resilience mechanisms are considered jointly.

**Table 4:** Development of the economic advantage of the resilience of the continuous cover system over the clear fell system under three scenarios, with severe disturbance either at stand age 60 (I) or at stand age 70 (II and III)

Time post disturbance	Economic resilience					
	(I) Economic return-based		(II) Structure-based		(III) Economic return- and structure-based	
	Advanced recovery [years]	Relative economic value of accelerated recovery [%]	Advanced recovery [years]	Relative economic value of accelerated recovery [%]	Advanced recovery [years]	Relative economic value of accelerated recovery [%]
	$\Delta r$	$\varOmega \cdot 100$	$\Delta r$	$\varOmega \cdot 100$	$\Delta r$	$\varOmega \cdot 100$
10	4.5	6.4	6.3	8.9	11.1	15.2
20	7.3	10.3	8.8	12.3	13.8	18.9
30	9.1	12.7	7.4	10.4	16.9	22.3
40	11.6	15.8	–	–	19.5	25.2

### Impact of Enhancing the Discount Rate on Resilience

Increasing the discount rate to $i=0.025$ reduces forest values roughly by 1/3 relative to those obtained for the default discount rate ($i=0.015$). In 30 years before disturbance the forest values of both silvicultural systems hardly differ. Under the higher discount rate the contribution of new tree cohorts to the total forest value decreases from about 45% (default discount rate 1.5%) to only 28% (discount rate 2.5%), as shown in Table 5 (“Appendix 3”) for 50-year old stands. Under the reduced contribution of the new tree cohorts, the current crop trees clearly dominate the forest value, which show a similar present value in both systems (under the elevated discount rate). Here the present value of the current crop trees is only 2.7% greater in the continuous cover system than in the clear fell system (for the example in Table 5). Thus, the total forest values of both systems are very similar.

**Fig. 7:**
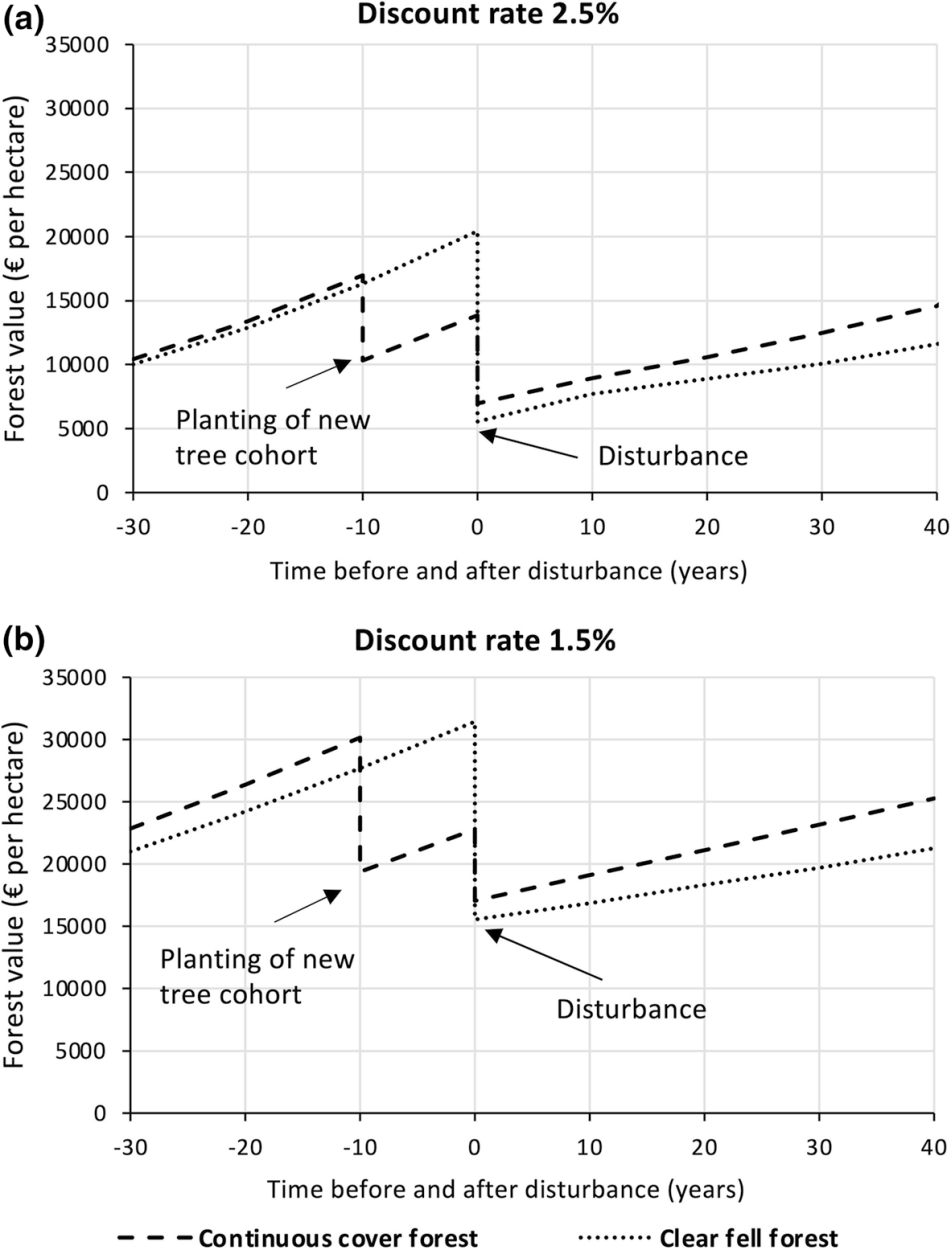
Influence of the discount rate on the economic resilience. **a** Pre- and post-disturbance development of forest value under both silvicultural systems when using an elevated discount rate of $i=0.025$ compared with **b** the development obtained under the default discount rate

**Fig. 8:**
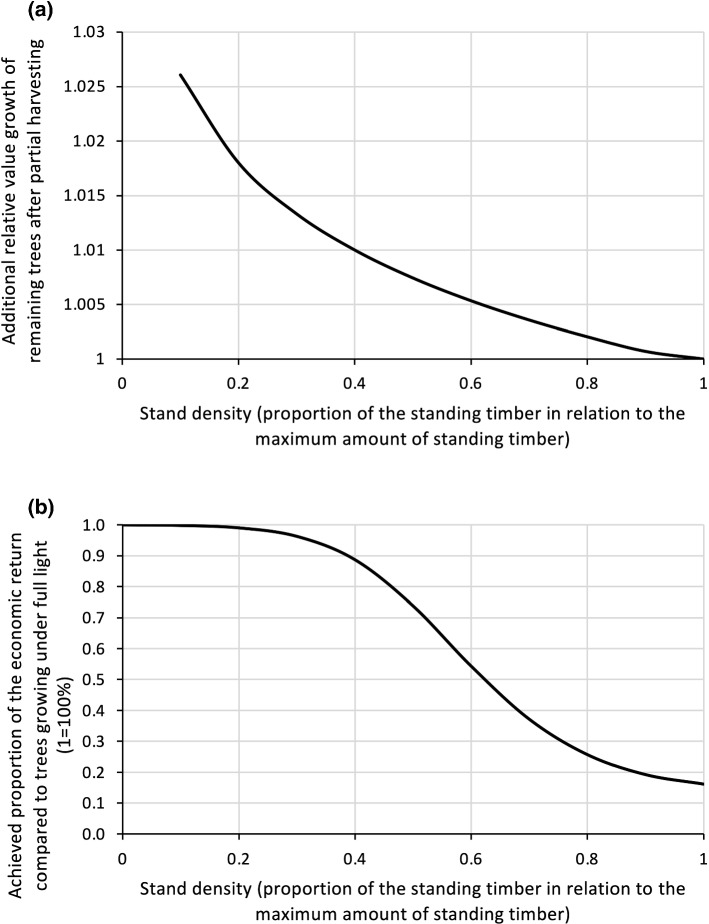
**a** Factors to simulate the relative increase of the value growth of remaining trees after partial harvest operations [based on data used in Härtl et al. (2010)]. **b** Achieved economic return of young trees when growing in gaps in relation to the economic return of trees growing in full light [reduction based on information provided by Kateb (2006) and Petriţan et al. (2009)], both variables depending on the stand’s density

While the pre-disturbance forest values of both forest systems are similar, their post-disturbance development shows a clear advantage for the continuous cover system, resembling the pattern which resulted under the default discount rate (Fig. 7).

The continuous cover system already achieves the same economic value 24 years after disturbance, which the clear fell system reached after 40 years of recovery (corresponding to an advantage of 16 years in system A). The accelerated recovery time of system A is thus slightly smaller compared to analyses using the default (lower) discount rate, where the advantage was almost 20 years. Still, the accelerated recovery of the continuous cover system under the higher discount rate corresponds to an economic gain in net present value of almost 33%. In relative terms, the economic advantage of the resilience of continuous cover forestry is thus even more pronounced under a higher discount rate.

## Discussion

### Revisiting the Hypotheses

Based on our application example, we found support for the hypothesis of higher economic return of continuous cover forestry compared to a clear fell system (hypothesis 1). Furthermore, we also found higher economic resilience of the continuous cover system, in part related to the establishment of a cohort of tree regeneration alongside the crop tree cohort (hypothesis 2). The higher economic return of the continuous cover system is inter alia the result of a positive growth response of trees to partial harvesting. Furthermore, also the establishment of young trees in gaps created by partial cuts contributes to the economic value of the stand. Compared to regular heavy thinning interventions in clear fell systems (which do not lead to tree regeneration) no production area is lost in these gap cuts, with favorable economic effects. Considering economic resilience, we show that beyond differences in economic return, resilience also depends on pre-disturbance forest structure. Our results thus confirm that continuous cover systems are advantageous over clear fell systems in at least two regards, first, by providing higher economic return, and second by higher economic resilience to severe disturbance.

Our results concerning hypothesis 1 are in line with those obtained with more sophisticated and complex non-linear optimization models. For example, the studies by Rämö and Tahvonen (2015), Roessiger et al. (2016), Assmuth et al. (2018), Parkatti and Tahvonen (2020), as well as Malo et al. (2021) support the conclusion that continuous cover systems may show economic superiority. This is aptly expressed by Tahvonen and Rämö (2016), stating that continuous cover forestry “… may not be overly expensive …; as a matter of fact, the reverse seems to be true …”. A convincing feature of these more sophisticated models is their ability to derive optimal forest management endogenously; underlining that continuous cover forestry may emerge when maximum net present value is the aim. Increasing discount rates and the availability of cost-efficient establishment of young trees through natural regeneration add to the economic attractiveness of continuous cover forestry. In our study, we have chosen a conservative approach by assuming young trees to be planted in both systems, disregarding another potential economic advantage of continuous cover forestry. While our model does not provide as nuanced analytical capacities as the ones mentioned above, it is still able to reproduce some of the key results of these previous studies, for example regarding the importance of the enhanced growth of remaining trees after partial harvest. Furthermore, while risks and uncertainties are commonly not part of advanced optimization approaches [but see Malo et al. (2021)] we here have integrated survival probabilities and the resulting effects of background mortality into the analysis of optimal forest management [see also Roessiger et al. (2011, 2013) and Messerer et al. (2017)]. Overall, we thus conclude that our model is a useful tool for studying the economic resilience of different silvicultural systems.

### Role of the Discount Rate

Although the recovery time of the continuous cover system increased under a higher discount rate, the relative advantage over the clear fell system was more pronounced under the higher discount rate compared to the lower discount rate (33% vs. 25% gain in the forest’s present value). This result supports the notion that continuous cover systems become more attractive with higher discount rates. This influence of the discount rate has so far only been demonstrated by studies that disregarded economic resilience. For example, Tahvonen (2015) demonstrated how higher discount rates can favor continuous cover forestry. His study provided several reasons for this effect, including a positive effect of not clearing small trees (which are usually less valuable) and a long delay between harvesting operations which is common in the clear fell system. In our study the economic advantage of the continuous cover system over the clear fell system increased from + 6% (discount rate $$i=0.005)$$ to + 16% ($$i=0.03$$). This influence of the discount rate was less pronounced in previous studies. Tahvonen (2009), for example, reported a more or less constant relative advantage of the continuous cover system for two levels of the discount rate, with + 32% ($$i=0.002$$) and + 30% ($$i=0.03$$).

A tendency for an increase in the relative size of the economic advantage of the continuous cover system with increasing discount rates appears plausible, when comparing the accelerated growth in economic value of remaining trees after density reduction (independent of the discount rate) with the opportunity costs of the clear fell system (dependent on the discount rate). Messerer et al. (2020), for example, quantified the economic advantages of accelerated growth of remaining trees after harvest operations. The opportunity costs of the clear fell system include holding a higher capital than under the continuous cover system that is tied up in the standing timber, as well as postponing the plantation of young trees.

Applying Pressler’s condition for optimal timber harvesting (e.g., Knoke et al. 2020c) we can show the effect of opportunity costs. Pressler’s condition states that it is optimal to hold a forest for at least one further year, if its annual growth in stumpage value exceeds the opportunity costs needed to facilitate the annual value growth. Opportunity costs are represented by the capital tied up in the stand’s standing timber and the opportunity costs of postponing future tree generations, expressed by the WPL for forestland considering a newly established forest. We can use Pressler’s condition to compare the effect of a gap harvest operation in the continuous cover forest with the growth in stumpage value of the fully stocked even-aged forest, considering the additional opportunity costs of the clear fell forest. For example, establishing a gap of relative size $g$ to initiate the transition towards continuous cover forestry and planting young trees at time $t$, when the standing timber has achieved a stumpage value of ${h}_{t}$, is advantageous, if the following condition holds

$${\dot{h}}_{t}^{{\prime}}\cdot \left(1-{g}_{t}\right)>{\dot{h}}_{t}-\left({h}_{t}+{L}_{\omega }^{{\prime}}\right)\cdot {g}_{t}\cdot i$$

where ${\dot{h}}_{t}^{{\prime}}$ is the annual growth of the stumpage value of the remaining continuous cover trees, accelerated as a consequence of the reduced stand density. In contrast, ${\dot{h}}_{t}$ is the annual growth of the stumpage value of the trees in the fully stocked clear fell forest, and $\left({h}_{t}+{L}_{\omega }^{{\prime}}\right)\cdot {g}_{t}\cdot i$ is the additional opportunity cost of the clear fell forest compared to the forest allowing for the gap harvestings. Here, ${h}_{t}$ is the financial value of the standing timber and ${L}_{\omega }^{{\prime}}$ is the anticipated economic return of future tree generations.

Our simulations imply ${\dot{h}}_{t}^{{\prime}}>{\dot{h}}_{t}$ as a consequence of the density reduction, while the area on which crop trees can produce timber is reduced to $1-{g}_{t}$ in the continuous cover forest. Reducing the area stocked with crop trees decreases the capital tied up in continuous cover trees so that the clear fell system holds higher capital, which may be described by ${h}_{t}\cdot {g}_{t}$, while ${h}_{t}\cdot {g}_{t}\cdot i$ is the additional opportunity costs of holding this capital. In addition, the clear fell system does not plant young trees with a bare land value of ${L}_{\omega }^{{\prime}}\cdot {g}_{t}$, resulting in further opportunity costs of ${L}_{\omega }^{{\prime}}\cdot {g}_{t}\cdot i$. From this it is clear that the opportunity costs of the clear fell system will increase with an increasing discount rate $i$, while the increase of the stumpage value (${\dot{h}}_{t}^{{\prime}}$) in the continuous cover system does not depend on the discount rate. This makes continuous cover forest economically more attractive under higher discount rates. The commonly disregarded delayed recovery of the clear fell system after severe disturbances adds to the opportunity costs of clear fell forestry and makes continuous cover forests even more attractive.

### Limitations

We have assessed economic resilience to a severe natural disturbance event, yet the probability for such an event to occur can vary widely. In Central Europe, for instance, the historical return interval of such events lies between approximately 150 and 700 years, depending on site conditions, management regimes, and other parameters (e.g., Thom et al. 2013; Janda et al. 2017). However, distributions of return intervals are usually skewed so that severe natural disturbance will affect some areas more frequently than others (Thom et al. 2013). Considering extreme events probabilistically in economic analyses thus remains difficult, because records for these events remain rare in observational data (Knoke et al. 2021). A further element of our work that needs consideration when interpreting the results is the fact that we considered disturbance effects only on crop trees but not the tree cohorts in the regeneration layer. For the prevailing natural disturbance regime in Central Europe (consisting mainly of wind and bark beetle disturbances, which tend not to affect trees in early development stages) this assumption is realistic. For other disturbance agents, such as wildfire, that also affect regenerating trees, our analysis framework would need amendments to reflect the different impact of disturbance. In addition, even in the context of wind and bark beetle disturbance our assumption of complete survival of the regeneration layer is optimistic. In reality, these disturbances and the subsequent salvage operations also result in some damage to young tree cohorts, a fact we did not consider here.

Our optimization model relies on several simplifying assumptions. For instance, we only consider one tree species (Norway spruce), and exclude the effects of mixing different tree species at the stand level. Accounting for possible effects of mixed forests, which can be beneficial in both continuous cover (Parkatti and Tahvonen 2020) and clear fell systems (Paul et al. 2019) would change the optimization results.

Given our simplified optimization approach, we have conducted extensive tests evaluating the robustness of our results to changes in the assumptions made (cf. Kuorikoski et al. 2010). For example, we tested the effect of a shorter, economically suboptimal rotation period for crop trees in the clear fell system (e.g., 60 instead of 70) (“Appendix 4”). While this assumption reduces the advantage of the continuous cover system, the latter still has higher overall resilience compared to the clear fell system. The effects of other important assumptions on the outcome of our analysis can be found in “Appendix 5” (Table 6). The conclusion from these sensitivity tests was that the economic advantage of the continuous cover system over the clear fell system remains largely robust. One exception to this finding is if the simulated growth of young trees growing in gaps in the continuous cover system is less than 50% of the currently assumed growth of trees in gaps. Such reduced growth performance will lead to higher economic return in the clear fell system compared to the continuous cover system (see Table 6).

## Conclusion

Continuous cover forestry is a tangible alternative to clear fell systems that remain dominant around the globe (Kuuluvainen et al. 2012; Puettmann et al. 2015). Previous analyses have suggested that continuous cover systems are ecologically resilient (i.e., able to recover quickly from disturbance) and resistant (being able to withstand disturbance) systems (O'Hara and Ramage 2013; Hanewinkel et al. 2014; Diaci et al. 2017). We here show that continuous cover systems also are more resilient than clear fell systems in economic terms. Our analyses suggest that continuous cover forestry could be an important approach for addressing increasing disturbances in forest management, buffering the detrimental economic impacts of climate-induced extreme events. Our framework on quantifying economic resilience has the potential to inform economic resilience research beyond questions of forest management.

## Figures and Tables

**Fig. 9:**
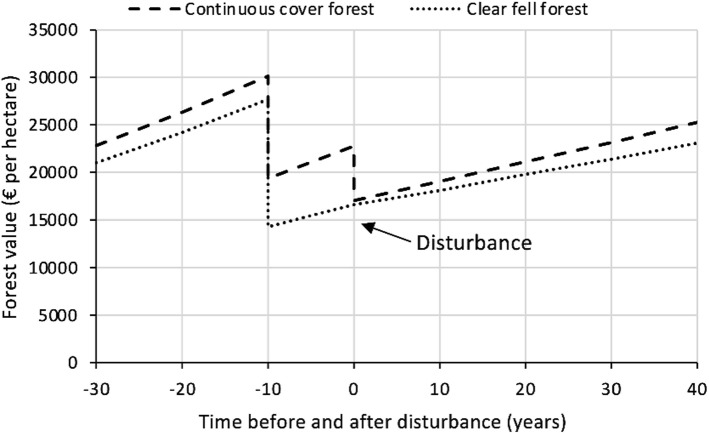
Harvest of the benchmark forest system already at an age of 60 (instead of 70 as computed optimum), which avoids the loss of the crop trees 10 years later (we assume that the 10-year old young stand remains unaffected by the disturbance)

## Appendix 1: Optimization Model to Maximize Economic Return

### The Benchmark Forest System

We formalize the optimization of our benchmark forest as:

$$\begin{aligned}&\underset{T}{\mathrm{max}}\,{R}_{T}\\ &{\mathrm{s.t.}}\,\,L_{T}:={R}_{T}\\ &{R}_{T}=-{C}_{0}+{C}_{T}+{D}_{T}\qquad({\mathrm{see}}\,{\mathrm{Eq.}}\, 14\,{\mathrm{main}}\,{\text{text}})\end{aligned}$$

${R}_{T}$: economic return of the benchmark forest system [all values here in € per hectare], when starting from bare ground, considering background mortality events; ${L}_{T}$: anticipated economic return of future tree generations, newly planted after a disturbance event on the salvaged areas or on regularly harvested areas, given a forest stand under the benchmark management system depending on *T*; i.e. the sum of all discounted net revenues anticipated over an unlimited time; ${C}_{0}$: establishment costs; ${C}_{T}$: present value of all net revenues from crop trees and from all trees planted after the harvest of crop trees over an unlimited time, on the proportion of a forest’s area surviving until *T*; ${D}_{T}$: present value of all net revenues accumulated over *T* associated with salvage logging due to background mortality events, including the economic return from all young trees planted on the salvaged areas over an unlimited time.

The present value of the proportion of our forest trees surviving until *T* follows from Eq. (22):

22 $${{C}_{T}=S}_{T}\left({h}_{T}+{L}_{T}\right){\beta }^{T}$$

${S}_{T}$: survival probability used to model the proportion of a forest’s area covered by crop trees of a maximum density surviving until *T* (maximum age or rotation period), driven by background mortality events; ${h}_{T}$: Stumpage value of a forest area under the benchmark forest system at *T*; stumpage value is the net value obtained from marketing timber, less of logging costs [€ per hectare]; ${L}_{T}$: anticipated economic return of future tree generations, newly planted after a disturbance event on the salvaged areas or on regularly harvested areas, given a forest stand under the benchmark management system depending on *T*; i.e. the sum of all discounted net revenues anticipated over an unlimited time [€ per hectare]; $\beta$: discount factor, $\beta =\frac{1}{1+i}$, with $i=0.015$ being our default discount rate; $T$: rotation period; time from establishment until final harvest of the crop trees.

The area proportion covered by crop trees surviving until *T* (${S}_{T}$) is the area on which we can harvest the stumpage value (${h}_{T}$), which is the net revenue expected when felling the standing timber (price per cubic meter times timber volume less harvest costs).

The discounted net revenues associated with the expected background mortality events enter Eq. (23). Similarly as in Eq. (22) we account for all net revenues after salvage logging due to background mortality events of a reduced stumpage value ($\gamma {h}_{t}$) by ${L}_{T}$.

23 $${D}_{T}=\sum_{t=0}^{T-l}{S}_{t}{\pi }_{t}\left(\gamma {h}_{t}+{L}_{T}\right){\beta }^{t}$$

24 $${S}_{t}={S}_{t-l}\left(1-{\pi }_{t-l}\right)$$

25 $${S}_{T}+\sum_{t=0}^{T-l}{S}_{t}{\pi }_{t}=1$$

${S}_{t}$: survival probability modelled as the proportion of a forest’s area covered by crop trees of a maximum density surviving until *t* (quantifying the background mortality); $l$: size of time steps considered; 10 years in our study; $t$: time in years; ${\pi }_{t}$: hazard rate at *t*, ${\pi }_{t}=\frac{{S}_{t}-{S}_{t+l}}{{S}_{t}}; \gamma$: proportion of the common stumpage value we can still achieve for damaged timber after a disturbance event; by default we use $\gamma =0.5$ to consider background mortality events, when simulating a severe disturbance event we set $\gamma =0$.

### The Alternative Forest System

We formalize the optimization of our alternative forest system as:

26 $$\begin{aligned} & \mathop {\max }\limits_{\omega } R_{\omega } \\ & {\text{s.t.}}\;\;L_{\omega } : = R_{\omega } \\ & R_{\omega } = - C_{0} + C_{H} + G_{\omega } + D_{H} \quad\;\;({\text{see}}\;{\text{Eq.}}\;15\;{\text{main}}\;{\text{text}}) \\ \end{aligned}$$

${R}_{\omega }$: expected economic return of the alternative forest system A, considering background mortality events [all values here in € per hectare]; ${C}_{0}$: establishment costs; ${ L}_{\omega }$: economic return of future tree generations managed exactly identical as the whole alternative forest system; ${C}_{H}$: present value of all net revenues from crop trees and trees planted after the harvest of crop trees over an unlimited time on the proportion of a forest’s area surviving until *H*; ${G}_{\omega }$: present value of partial harvests consisting of all discounted net revenues from crop trees harvested in gaps and from young trees planted after the harvest of crop trees over an unlimited time; ${D}_{H}$: present value of all net revenues accumulated over the horizon *H* associated with salvage logging due to background mortality events, including the economic return from all young trees planted on the salvaged areas over an unlimited time.

In the case of the alternative forest system the optimization consists of searching for the optimal set of area proportions of partial harvests $\omega =\left\{{g}_{t=z};\dots ;{g}_{H-l}\right\}$, comprising the sizes of gaps created to establish young trees, allocated to different times *t*. The allocation of gaps of variable sizes over the horizon *H* has a direct impact on ${G}_{\omega }$. For the optimization we used Frontline Systems’ Analytic Solver (V2020), using the Standard GRG nonlinear Engine. We needed a nonlinear solver, as the simulated growth response of the remaining crops trees and of the young trees planted in the created gaps after partial harvests made the optimization problem nonlinear.

${C}_{H}$ in our objective function represents the present value of the last cohort of crop trees harvested at the end of the horizon *H*.

27 $${C}_{H}={S}_{H}^{u}\left({h}_{H}^{{\prime}}+{L}_{\omega }\right){\beta }^{H}$$

${S}_{H}^{u}$: survival probability modelled as the proportion of a forest’s area surviving until *H* and still not covered by young trees planted during previous gap-cut operations; ${h}_{H}^{{\prime}}$: stumpage value of a forest under the alternative management system at *H*, depending on the density of the forest stand at each *t* before reaching *H* [€ per hectare]; ${L}_{\omega }$: economic return of future tree generations planted under the alternative management system, depending on the distribution of gap harvestings over time symbolized by $\omega$.

${S}_{H}^{u}$ is different to the survival probability ${S}_{T}$ in Eq. (23), which is necessary to account for the gaps created by our simulated partial harvests. ${S}_{H}^{u}$ follows from:

28 $${S}_{H}^{u}={S}_{H-l}^{u}\left(1-{g}_{H-l}\right)\left(1-{\pi }_{H-l}\right)$$

${S}_{H-l}^{u}$: proportion of a forest’s area surviving until *H–l* and still not covered by young trees planted during previous gap-felling operations; ${g}_{H-l}$: relative size of a gap established at *H–l*; ${\pi }_{H-l}$: hazard rate at *H–l.*

Core component of the alternative management system is an estimation of the present value of harvesting of crop trees to establish young trees in gaps prior to the end of the horizon *H* (Eq. 28). In addition to the net revenues from harvesting crop trees, the economic return from future tree generations ${L}_{\omega }^{{\prime}}$ accounts for all discounted net revenues of the young trees planted in the gaps.

29 $${G}_{\omega }=\sum_{t=z}^{H-l}{g}_{t}\left({S}_{t}{h}_{t}^{{\prime}}+{S}_{t}^{u}{L}_{\omega }^{{\prime}}\right){\beta }^{t}$$

The survival probability ${S}_{t}^{u}$ depends on the sizes of the established gaps (${g}_{t}$).

30 $${S}_{t}^{u}={S}_{t-l}^{u}\left(1-{g}_{t-l}\right)\left(1-{\pi }_{t-l}\right)$$

$z$: time when simulating the first gap felling and subsequent establishment of young trees, for which we assume a minimum age of 30; ${g}_{t}$: size of a gap established at *t* (e.g., 0.15 for 0.15 hectares per hectare) either computed as a proportion of ${S}_{t}$ (stocked with timber already reduced by previous gap-harvest operations) to obtain the timber harvest or as a proportion of ${S}_{t}^{u}$ to receive the size of the newly planted tree cohort; ${S}_{t}^{u}$: proportion of a forest’s area surviving until *t* and still not covered by young trees planted during previous gap-felling operations; ${h}_{t}^{{\prime}}$: stumpage value of a forest under the alternative management system at *t*, depending on the harvesting operations and stand densities at all previous times *t* [€ per hectare]; ${L}_{\omega }^{{\prime}}$: sum of discounted net revenues of young trees planted in the forest gaps, used to quantify the economic return of all future tree generations [€ per hectare]. Compared with the economic return of young trees growing under full light, the economic return simulated for young trees under the alternative management system is lower, as long as they grow in gaps (see Fig. 8b).

A factor (${\delta }_{d}^{l}$) depending on the stand’s density facilitates to adjust the stumpage value (${h}_{t}^{{\prime}}$) under the alternative management system. Such adjustment accounts for growth responses of unharvested crop trees after reducing the stand density. ${\delta }_{d}^{l}$ follows from Eq. (31) (see Sect. 2.5):

31 $${\delta }_{d}^{l}=exp\left(-0.0005-0.0114{\mathrm{ln}}\,d\right)$$

$d$: density of the forest stand, proportion of the actually available standing timber volume in relation the maximally possible standing timber volume.

Based on ${\delta }_{d}^{l}$ we modeled ${h}_{t}^{{\prime}}$ as follows:

32 $${h}_{t}^{{\prime}}={h}_{t-l}^{{\prime}}{\delta }_{t}^{l}{\delta }_{d}^{l}$$

${\delta }_{t}^{l}$: growth factor at *t*, ${\delta }_{t}^{l}=1+w$, with *w* being the relative value growth under the maximum stand density (i.e. value growth percent) and accumulated over *l*, the time step considered (i.e. 10 years); ${\delta }_{d}^{l}$: density dependent factor of growth increases, ${\delta }_{d}^{l}=1+avg$, with *avg* being the additional relative value growth when the stand’s density declines relative to the maximum stand density so that the remaining trees can grow faster.

We finally adjust ${L}_{\omega }^{{\prime}}$, the economic return of the young trees planted in the forest gaps, according to Eq. (34). The adjustment adopts the factor ${\vartheta }_{d}$ to quantify the proportion of the economic return achieved in a gap, under reduced light availability. ${\vartheta }_{d}$ is (see Sect. 2.5 for the information sources used):

33 $${\vartheta }_{d}={\left[1+exp\left(-7.7766465+0.1754335d-0.00081175{d}^{2}\right)\right]}^{-1}$$

For the economic return of future tree generations planted under the alternative management system, we write:

34 $${L}_{\omega }^{{\prime}}={\vartheta }_{d}{L}_{\omega }\left({\beta }^{-\left(H-t\right)}-1\right){\beta }^{H-t}+{L}_{\omega }{\beta }^{H-t}$$

${L}_{\omega }^{{\prime}}$: economic return of tree cohorts planted at *t*, for which the competition of the still existing crop trees reduces growth over the period *H–t*; ${\vartheta }_{d}$: density dependent proportion of maximum economic return achieved by a newly planted tree cohort accounting for the competition of the crop trees. ${\vartheta }_{d}$ varies between zero and 1.

Figure 8 shows how we modeled the growth increase of remaining trees after partial harvests (Fig. 8a) and the achieved economic return of young trees planted in gaps, relative to the economic return of trees growing in full light (Fig. 8b).

The consideration of the discounted net revenues associated with background mortality events is similar to the benchmark case, but accounts for the gaps established under the alternative system (Eq. 35).

35 $${D}_{H}=\sum _{t=0}^{H-l}{\left(1-{g}_{t}\right)}S_{t}^{u}{\pi }_{t}\left(\gamma {h}_{t}+{L}_{\omega }\right){\beta }^{t}$$

36 $${S}_{H}^{u}+\sum _{t=z}^{H-l}{g}_{t}{S}_{t}^{u}+\sum _{t=0}^{H-l}{\left(1-{g}_{t}\right)}S_{t}^{u}{\pi }_{t}=1$$

## Appendix 2: Economic Value of the Alternative Forest System

We obtained the economic forest value of the alternative forest system as:

$$\begin{aligned} & \max \;A_{a} \\ & {\text{s.t.}}\;F_{a} : = A_{a} \quad \forall a \\ & A_{a} = C_{a} + G_{a,\omega } + D_{a} + F_{a,\omega } + F_{a,D} \,\,\,\;\;\;({\text{see }}\;{\text{Eq.}}\,13\;{\text{main}}\;{\text{text}}) \\ \end{aligned}$$

${A}_{a}$: expected economic value of the alternative forest system A, considering background mortality events, but no extreme disturbance events [all values here in € per hectare]; ${F}_{a}$: forest value of the oldest tree cohort planted after the first simulated salvage logging; ${C}_{a}$: present value of all net revenues from crop trees remaining until *H* and trees planted after the harvest of the last crop trees, considered for a stand of age *a*, thus discounted over *H-a*; ${G}_{a,\omega }$: sum of present values associated with future partial harvests, consisting of all discounted net revenues from crop trees harvested to create gaps and from young trees planted after the harvest of crop trees over an unlimited time, for a stand of age *a*, so discounted over *t–a*; ${D}_{a}$: present value of all net revenues occurring from background mortality at any future *t*, considered for a stand of age *a*, so discounted over *t–a*; ${F}_{a,\omega }$: sum of forest values of tree cohorts planted after partial harvests in gaps at any *t* until reaching *a*; ${F}_{a,D}$: sum of forest values of tree cohorts previously planted on areas affected by background mortality events and salvaged in each period until *a*.

The component ${C}_{a}$ describes the value of the trees becoming oldest over the production horizon *H*.

37 $${C}_{a}={S}_{H}^{u}\left({h}_{H}^{{\prime}}+{L}_{\omega }\right){\beta }^{H-a}$$

We obtain the sum of the present values of future partial harvests from age *a* onwards as:

38 $${G}_{a,\omega }=\sum _{t=a+l}^{H-l}{g}_{t}\left({S}_{t}{h}_{t}^{{\prime}}+{S}_{t}^{u}{L}_{\omega }^{{\prime}}\right){\beta }^{t-a}$$

The present value of all net revenues resulting from disturbances at any future *t* follows from:

39 $${D}_{a}={\sum _{t=a+l}^{H-l}\left(1-{g}_{t}\right)}S_{t}^{u}{\pi }_{t}\left({\gamma h}_{t}^{{\prime}}+{L}_{\omega }\right){\beta }^{t-a}$$

In a further step, we obtain the sum of all forest values formed by trees already planted after previous partial harvests as:

40 $${F}_{a,\omega }=\sum _{t=z}^{a}{g}_{t}{S}_{t}^{u}{F}_{a-t}^{{\prime}}$$

$z$: time when simulating the first gap felling and subsequent establishment of young trees, for which we assume a minimum age of 30; ${F}_{a-t}^{{\prime}}$: forest value formed by a tree cohort already planted until *a* in gaps created by previous partial harvest operations; the forest value was reduced compared to that of trees grown in full light by multiplying ${F}_{a-t}$ with $L_{\omega }^{{\prime}} \div L_{\omega }$.

Finally, we compute the sum of all forest values formed by trees planted because of background mortality prior to achieving the stand age *a* on salvaged areas as:

41 $${F}_{a,D}=\sum _{t=0}^{a}{\left(1-{g}_{t}\right)}S_{t}^{u}{\pi }_{t}{F}_{a-t}$$

With:

42 $${S}_{H}^{u}+\sum_{t=a+l}^{H-l}\left[{g}_{t}{S}_{t}^{u}+\left(1-{g}_{t}\right){S}_{t}^{u}{\pi }_{t}\right]+\sum _{t=z}^{a}{g}_{t}{S}_{t}^{u}+\sum _{t=0}^{a}{\left(1-{g}_{t}\right)}S_{t}^{u}{\pi }_{t}=1$$

## Appendix 3: Contribution of Current Crop Trees and New Age Cohorts to the Forest Value, Established After Salvage Logging, Gap Harvests or Clear Felling

See Table 5.

**Table 5:** Composition of the forest value of a continuous cover and a clear fell forest for a 50-year old stand under two discount rates

	*Forest value in € per hectare*			
	Clear fell forest		Continuous cover forest	
	Discount rate			
	1.5%	2.5%	1.5%	2.5%

Element of forest value	Crop trees	New tree cohorts	Crop trees	New tree cohorts	Crop trees	New tree cohorts	Crop trees	New tree cohorts
${C}_{a}$	12,652	3892	11,338	1220	517	31	141	5
${D}_{a}$	1104	1079	497	160	696	905	230	92
${F}_{a,D}$		5508		3120		5989		3245
${G}_{a,\omega }$	Not applicable				13,299	4910	11,790	1483
**Sums**	**13,756**	**10,479**	**11,835**	**4500**	**14,512**	**11,835**	**12,161**	**4825**
Total forest value	24,235		16,335		26,347		16,986	
**Contribution**	**57%**	**43%**	**72%**	**28%**	**55%**	**45%**	**72%**	**28%**
${C}_{a}$	: present﻿ value of all net revenues from crop trees remaining until *H* or *T* and trees planted after the harvest of the last crop trees, considered for a stand of age *a*, thus discounted over *H–a* or over *T–a*							
${G}_{a,\omega }$	: sum of present values associated with future partial harvests, consisting of all discounted net revenues from crop trees harvested to create gaps and from young trees planted after the harvest of crop trees over an unlimited time, for a stand of age *a*, thus discounted over *t–a*							
${D}_{a}$	: present value of all net revenues occurring from background mortality at any future *t*, considered for a stand of age *a*, and therefore discounted over *t–a*							
${F}_{a,D}$	: sum of forest values of tree cohorts previously planted on areas affected by background mortality and salvaged in each period until *a*							

## Appendix 4: Reducing the Rotation Length to Enhance the Resilience of the Benchmark Forest System

A reduction of the harvest period from age 70 to age 60 does not does not improve the benchmark forest resilience compared to the alternative forest system, although the alternative forest suffers from the disturbance and the benchmark forest system does not (Fig. 9). Our consideration implies that the newly planted young stand remains unaffected by the disturbance.

## Appendix 5: Robustness of the Economic Return of the Alternative Forest System

See Table 6.

**Table 6:** Differences of economic return of the alternative forest system and the benchmark forest system

Changed variable	Degree of change	Difference of economic return [€ per hectare]
(1) Hazard rate after creation of gaps	+ 0%	1003
	+ 10%	906
	+ 20%	833
	+ 30%	758
	+ 40%	688
	+ 50%	624
	+ 60%	564
	+ 70%	509
	+ 80%	459
	+ 90%	412
	+ 100%	369
(2) Maximum level of timber harvest per decade [m^3^ per hectare]	70	− 614
	100	45
	150	429
	200	559
	250	894
	300	1001
	350	1003
(3) Survival probability up to age 100 [%]	20.8	559
	30.8	835
	40.8	1003
	50.8	1184
	60.8	1380
	70.8	1597
(4) Economic return of young trees while growing in gaps, here reduced by a change of ${\vartheta }_{d}$. ${\vartheta }_{d}$ is the density dependent proportion of the maximum economic return achieved by a newly planted tree cohort, accounting for the competition of the crop trees	− 0%	1003
	− 10%	779
	− 20%	568
	− 30%	371
	− 40%	187
	− 50%	15
	− 60%	− 144
	− 70%	− 287
	− 80%	− 368
	− 90%	− 469
	− 100%	− 556
(5) Response of the value growth to reductions in tree density, when 100% represents the default response	50%	231
	60%	356
	70%	497
	80%	652
	90%	822
	100%	1003
	110%	1193
	120%	1392
	130%	1599
	140%	1813
	150%	2034

A positive difference indicates a higher economic return of the alternative forest system, a negative difference stands for a higher economic return of the benchmark forest system
